# Markers of vulnerability in schizophrenia

**Published:** 2009-04-25

**Authors:** M Ladea, D Prelipceanu

**Affiliations:** ‘Prof. Dr. Al. Obregia’Clinical Psychiatry Hospital, Bucharest Romania

## Abstract

Vulnerability in schizophrenia is an integrative concept, which tries to explain the development of schizophrenia as an interaction between different individual susceptibility factors and environmental risk factors. Vulnerability markers used in genetic studies include biochemical indicators, neuroanatomical, neurophysiologic, and cognitive abnormalities. Among those, the most extensive studied markers were: evoked potentials, smooth pursuit eye movements, and attentional deficits. Some of the potential indicators presented in this paper satisfy most of the criteria necessary for a vulnerability marker, but none meets all of them. Nevertheless, they represent important markers of risk to schizophrenia.

**Key words:** vulnerability, evoked potentials, eye movements, attentional deficits

## Introduction

The studies of schizophrenia etiology are focused mostly on vulnerability models, an integrative concept, which has as main objective the explanation of the variability of experimental and clinical data. This bio–psycho–social approach considers that the development of schizophrenia is determined by the complex interaction between different factors and suggest explanatory hypothesis for both the etiology and the clinical variability. The concept of vulnerability describes the complex interactions between individual susceptibility factors and environmental risk factors. These interactions are at the origin of high risk or of the clinical symptomatology.

The clinical heterogeneity of schizophrenia and spectrum disorders creates important methodological difficulties for genetic studies. One solution for the research of genetic vulnerability for schizophrenia is the definition of endophenotypes, based on clinical, cognitive, or biological parameters (*Gottesman and Gould,* 2003). An efficient endophenotype should be reliable, stable, hereditary, and to identify the risk of an individual to develop the disease, as by example the high level of cholesterol indicates a risk for cardiovascular disease

*Graver *(1987) proposes the following criteria in defining a vulnerability marker or a trait marker, capable of detecting the biological risk to develop a psychosis: the different distribution in patients versus control group; higher prevalence in family members than in general population; association with spectrum disorders in family members; correlation with spectrum disorders in children with high risk and presence of the marker before the manifestation of  clinical symptoms; reliability and stability in time. Some of the markers meet most of the criteria, but none meets all of them.

The main advantage in using endophenotypes resides in their correlation with functional and structural abnormalities associated with schizophrenia, and thus the approach of genetic mechanisms becomes more accurate. The importance of endophenotypes and their use in the research of schizophrenia has been extensively discussed in the psychiatric literature (*Braft and Freedman,* 2002; *Braft et al.* 2007; *Matei and Davidson*, 2007a, b).

Vulnerability markers used in genetic studies include biochemical indicators, neuroanatomical, neurophysiological and cognitive abnormalities, which proved to have a significant heritability rate (*Szymanski et al.*, 1994). Among those, most studied are: ocular movements (*Peralta et al.*, 1992; *Greenwood et al.,* 2007; *Martin et al.,* 2007), evoked potentials (*Freedman et al.,* 1997; *Winterer et al.,* 2003; *Yeap et al.,* 2006;*Greenwood et al.,*2007; *Martin et al.,* 2007) and cognitive evaluations (*Cannon et al.,* 2005; *Greenwood et al.,* 2007; *Gur et al.,* 2007). Others endophenotypes are structural cerebral anomalies (*McDonald et al.,* 2004; *Cannon et al.,* 2005; *Gurling et al.,* 2006) and alterations of D2 dopaminergic receptors densities (*Hirvonen et al.,* 2005). All these markers constitute specific traits in patients with schizophrenia and in a significant proportion of their relatives, and thus they represent markers of risk to this illness.

## Electrophysiological anomalies

Electrophysiological methods do not offer data that may be considered specific in psychiatry. Although in patients with schizophrenia, electroencephalographic (EEG) registrations show numerous anomalies (*Morihisa et al.*, 1983;* Morrison–Stewart et al.*, 1991;* Gruzelier et al.* 1990; *Symond et al.*, 2005; *Ferrarelli et al.*, 2007;* Knyazeva et al.*, 2008), more studies are necessary to confirm their value as biological markers. However, EEG data correlate with those obtained by other methods: evoked potentials, polisomnography (*Maixner et al.*, 1998).

**Evoked potentials (EP)** represent the answer of nervous cells to sensorial (visual, acoustic, tactile) or cognitive stimuli. In the case of cognitive stimuli, during the registration, the subjects execute certain tasks or tests correlated with sensoria stimuli, and this is why they are also called **event related potentials (ERP)**. Evaluation of EP illustrates important anomalies in patients with schizophrenia, and though they do not have a high specificity, they are promising directions of research ( *Bruder et al.*, 1999; *Doniger et al.*, 2002;* Ford et al.*, 2004; *Butler et al. *, 2005; *Haenschel et al.*, 2007). It is important to emphasize that in patients with schizophrenia EP are heritable (*Young et al.*, 1996;* Hall et al.*, 2006).

One of the most studied is the **P50** evoked potential, which demonstrates abnormalities in patients with schizophrenia and spectrum disorders (*Clementz et al.*, 1998;* Cadenhead et al.*, 2000; *Siever and Davis* ,2004).

The cognitive psychophysiology research was focused on **P300** evoked potential, which seems to be correlated with information processing (*Ford et al.*, 2004). Schizophrenic patients show low amplitude of P300 and increase in response latency (*St Clair et al.*, 1989). The decrease of visual and auditive P300 amplitude could be correlated with negative symptoms (*Pfefferbaum et al.*, 1989), and increase of auditive P300 amplitude with positive symptoms (*Shenton et al.*, 1989). Observed anomalies and especially increase of latency reflects problems of attention and information processing

Impact of RRF upon volemic status and cardiac hypertrophy.

Another extended studied evoked potential in patients with schizophrenia and spectrum disorders is **N400**, correlated with semantic associations (*Nestor et al.*, 1997;*Niznikiewicz et al.*, 1999). 

It is important to emphasize that the observed abnormalities are not specific. Anomalies of P300 were seen in patients with other psychiatric conditions, such as borderline personality disorder (*Blackwood et al.*, 1986; *Kutcher et al.* , 1987) or dementia (*Goodin et al.*, 1978). Changes of P50 wave were also found in patients with bipolar or schizoaffective disorder (*Martin et al.*, 2007). The results of the studies concerning EP are thus variable and more research is needed in this area

The amount of data brought by electroencephalography and evoked potentials is impressive. The quantitative analysis and topographical EEG (*Gruzelier et al.*, 1990) are advanced methods, assisted by computer, which makes the interpretation of data much easier.

*Karson et al.* (1988) apply **quantitative EEG (QEEG)** in untreated patients with schizophrenia and describe a slow alpha rhythm associated with enlargement of lateral cerebral ventricles. QEEG associated with cognitive tests enables the analysis of answers to different tests and tasks, at cerebral level. These methods have limited use and they do not have a clear clinical specificity. One of the applications of quantitative electrophysiology is the evaluation of effects for different drugs. The studies are based on the hypothesis that the substance has a certain impact on behavior and therefore will determine a specific electrophysiological activity, measurable by EEG. The research in this domain demonstrates that the main classes of psychoactive substances determine characteristic changes in the spectrum of EEG frequencies, even when administrated in acute situations. 

## Smooth pursuit eye movements

**Smooth Pursuit Eye Movements** (SPEM) were extensively investigated because they are under genetic control and constitute a biological marker that could define a vulnerability to schizophrenia and spectrum disorders (*Lee and Williams,* 2000). The studies are focused also on the relationship between SPEM and other dysfunctions of eye movements (saccades) observed in patients with schizophrenia (*Haraldsson et al.*, 2008). The physiopathology of SPEM seems to be associated with complex anomalies, especially in frontal area, but also in temporal and cingular areas, but more studies are needed in this direction (*Lee and Williams*, 2000; *Tregellas et al.*, 2004; *Hong et al.*, 2005; *Nagel et al.*, 2007).

SPEM dysfunctions were described in subjects with schizophrenia by numerous authors (*Moser et al.*, 1990;* Abel et al.*, 1991;* Friedman et al.*, 1991, 1992; *Hommer et al.*, 1991). They are found in about 50-80% of patients with schizophrenia versus 10% of control subjects (*Clementz and Sweeney,* 1990). The degree of dysfunction seems to be significantly greater in patients with schizophrenia than in control subjects (*Holzman et al. *, 1984; *Holzman,* 1987). The abnormalities are present in patients in both acute and remission phases, as well as in chronic patients (*Cegalis and Sweeney,* 1979; *Iacono et al.*, 1981; *Bartfai et al.* , 1985;* Rea et al.*, 1989), without specificity for a certain subgroup of schizophrenias (*Shagass et al.* , 1974). Nevertheless, some authors identify an association between disorganization syndrome and SPEM anomalies (*Lee et al. *, 2001). 

*Ross *(2003) emphasizes that SPEM dysfunctions may be identified even in childhood, in subjects with vulnerability to schizophrenia, in concordance with the neurodevelopment theory.

In order to avoid the influence of antipsychotic treatment, most authors compared untreated patients (on short periods) with treated patients, without being able to find differences between the two groups (*Holzman et al.*, 1974; *Siever et al. *, 1986; *Litman et al.*, 1989). Introducing the treatment in untreated patients does not have a significant impact on results (*Levy et al.*, 1983; *Kufferle et al.*, 1990).* Campion et al.* (1992) s tudy a group of never treated patients with schizophrenia and demonstrate that observed dysfunctions do not differ in a significant manner, from those in chronic patients under treatment.

Changes in SPEM are not specific to schizophrenia (*Kathmann et al.*, 2003). They are also found in individuals with spectrum disorders (*Siever et al.*, 1990), and bipolar disorder (*Shagass et al.*, 1974, *Lipton et al.*, 1980; *Iacono et al.*, 1982), but in this last group, the treatment with lithium seems to be responsible for changes in SPEM (*Levy et al.*, 1985; *Holzman et al.*, 1991).

Overall, the studies indicate the presence of a significant familial aggregation for SPEM dysfunctions and thus of genetic implication (*Kathmann et al.*, 2003; *Hong et al.*, 2006). SPEM abnormalities were identified in 30–50% of relatives of patients with schizophrenia that present these anomalies (*Holzman et al.*, 1974, 1984;* Mather *, 1985),  in comparison with only a small percentage of relatives of patients with other disorders. Some of these studies evaluate the clinical status of relatives. The patients with schizophrenia and their relatives with spectrum disorders present significant changes in SPEM in comparison with healthy relatives or control group (*Clementz et al.*, 1990;* Clementz and Sweeney*, 1990). *Blackwood et al.* (1991) demonstrated the presence of SPEM anomalies for an important percentage of relatives (without clinical manifestations) of patients with schizophrenia.

As for the study of twins discordant for schizophrenia, *Holzman et al.* (1977) found a high concordance, of 80%, for these anomalies in monozygots and a concordance of 39% for dizygots. Data illustrate the important role of genetic factors (*Matthysse et al.*, 1986; *Holzman et al.*, 1988;* Grove et al.*, 1992).

In the studies of relatives of patients with schizophrenia, SPEM anomalies contribute to the description of an enlarged clinical spectrum that includes subjects without symptoms. In other words, these changes could constitute a possible manifestation of a genetically determined latent trait, the symptoms of schizophrenia being only one of the possible phenotypical expressions of this trait. The marker is useful in genetic linkage studies.

In conclusion, SPEM abnormalities are a biological trait marker, but not a test that could confirm the diagnosis of schizophrenia ( *Szymansky et al.*, 1994).

## Changes in the electrodermal activity

The electrodermal activity is studied for a long time, and the results show that 40-50% of patients with schizophrenia present abnormalities, versus 5–10% of control group subjects (*Holzman*, 1987). The change of electrodermal answer is not specific to schizophrenia and so it is found in other groups of patients as well, especially those with affective disorders. Variable results were obtained when trying to correlate the changes in electrodermal activity with the symptomatology (*Straube*, 1979;* Bernstein et al.*, 1981;* Öhman*, 1981;* Alm et al.*, 1984;* Dawson and Nuechterlein*, 1984;* Green et al.*, 1989; *Williams et al.*, 2003). One of the research areas is the lateralization phenomenon, because a left-right asymmetry was found in the electrodermal activity (*Bob et al.*, 2007a, b).

The electrodermal answer constitutes a biological marker whose potential value justifies more in deep research (*Dawson et al. *, 1992), the results up to date showing that electrodermal anomalies are frequently associated with a poor outcome regarding both the symptoms and the social insertion (*Dawson and Schell*, 2002;* Schell et al.*, 2005).

## Cognitive impairement

MAttentional deficits are among the most promising vulnerability markers. Patients with schizophrenia have low performances in numerous neurocognitive tests, which evaluate different aspects of attentional processes. Only some of these aspects satisfy sufficient criteria necessary for a vulnerability marker.

The most accepted test for attentional deficits is the** Continuous Performance Test** (CPT;*Rosvold et al.*, 1956), which presents alterations independent of the stage of the illness (*Orzack and Kornetsky*, 1971; *Nuechterlein et al.*, 1992;* Cornblatt and Keilp*, 1994). Thus, attentional deficits evaluated with CPT, especially the problems related to the focusing of attention, are considered a good neurophysiological indicator for the risk of developing schizophrenia. Between 40 and 50% of patients with schizophrenia, show low performances in CPT (* Erlenmeyer–Kimling and Cornblatt*, 1987). Treatment may improve results of patients with schizophrenia, but these remain inferior to those obtain by the control group (*Harvey et al.*, 1990; *Serper et al.*, 1990;* Earle–Boyer et al.*, 1991). Nevertheless, there are studies that do not confirm the up–mentioned results (* Finkelstein et al.*, 1997;* Addington and Addington,* 1997).

*Cornblatt and Keilp* (1994) emphasize that attentional deficits are not only present independent of the patients' clinical status but are also detectable before the onset of illness and seem to be hereditary, observation confirmed by other studies. Decreased performances at CPT were also registered in subjects with high risk for schizophrenia (*Nuechterlein,* 1983;* Nuechterlein et al.*, 1986;* Erlenmeyer–Kimling and Cornblatt*, 1987;* Goldberg et al.*, 1990; *Lezenweger et al.*, 1991;* Maier et al.*, 1992;* Franke et al. *, 1994;* Chen et al.*, 1998), and these findings support the genetic implications of attentional deficits.

In conclusion, although in schizophrenic patients different other attention tests were applied, CPT remains the most useful and recognized.

Among the cognitive impairments of patients with schizophrenia, considered good candidates as vulnerability markers, are the low performances in tests sensitive at the change of frontal functions, especially Wisconsin Card Sorting Test (WCST; Berg, 1948). The impairments at this test (*Franke et al.*, 1993,* Lezenweger and Korfine*, 1994) support the hypothesis of the role played by the frontal area in the vulnerability to schizophrenia. Functional imagistic procedures, as well as electroencephalographic ones, applied during the execution of cognitive tests support this correlation (*Buchsbaum et al.* , 1990;* Hoffman et al.*, 1991;* Mann et al.*, 1997), the results strongly suggesting the role of prefrontal area in the vulnerability for schizophrenia. 

Research of cognitive and behavioral markers include evaluations of neurobehavioral performances (motor, visual–motor, attentional, cognitive) as well as the evaluation of social functioning, organizing capacity, intelligence, individual autonomy in subjects at risk but also in apparently healthy young individuals (*Davidson et al.*, 1999;* Hans et al.*, 1999;* Gur et al.*, 2007). These indicators are usefully in identifying the risk to develop schizophrenia.

## Neuro–psycho–endocrinological anomalies

The main hypotheses of the role of hormonal changes in schizophrenia (*Lembreghts and Ansseau*, 1993; *Liberman and Koreen*, 1993) are illustrated by the studies of the growth hormone and the hypothalamic–pituitary–adrenal system, and especially the interactions of these with the neurotransmitters (*Bennett*, 2008;* Pruessner et al.*, 2008; *Walker et al.*, 2008). The relation between cortisol and glutamate, and cortisol and dopamine were more studied, but the results are inconclusive

More important seems to be the estrogenic hypothesis, which is correlated with later onset of illness, better premorbid functioning and better outcome of schizophrenia in women than in men. Estrogen impact upon central nervous system is studied through the interactions with: dopamine (*HȨfner et al.*, 1991;* Bossé and Di Paolo*, 1996), serotonin (*Bossé and Di Paolo *, 1996;* Sumner and Fink*, 1995;* Fink et al.*, 1996;* McQueen et al.*, 1997), gamma–amino–butyric acid (*Bossé and Di Paolo*, 1996), and glutamate (*Gazzaley et al.*, 1996;* Diano et al.*, 1997). This is one of the explanatory models of the variability of schizophrenia in women versus men and it might have some therapeutic implications (*Kulkarni et al.*, 2001, 2008).

## Immune markers

The hypothesis of an immune dysfunction was approached from different perspectives. One of the most interesting concepts refers to the implication of autoimmune mechanisms (*Goldsmith and Rogers*, 2008), but the results do not allow significant conclusions

The most frequent modifications observed include interleukins, lymphocytes, antinuclear antibodies, which suggest that in the etiology of schizophrenia immune abnormalities could play an important role (*Müller et al.*, 1999, 2000;* Printz et al. *, 1999;* Tanaka et al.*, 2000).

The hypothesis of a viral infection during pregnancy in women that gave birth to a child, that later developed schizophrenia, is still controversial.

##  Conclusions

Biological markers have a great impact upon genetic studies because they are quantifiable measures that can reduce the heterogeneity specific to psychiatric disorders and especially to schizophrenia. These markers may be of neuropsychological, neuroanatomic, electrophysiological nature. Not all described markers have the same importance in research. The most useful are, for now, the dysfunctions of evoked potentials and eye movements and the attention deficits
